# Supplementary material for: Diversity and plant growth promoting ability of rice root-associated bacteria in Burkina-Faso and cross-comparison with metabarcoding data
Source: PLoS One. 2023 Nov 30;18(11):e0287084. doi: 10.1371/journal.pone.0287084 (PMC10688718; doi:10.1371/journal.pone.0287084)
Supplement: S1 Fig — (PPTX) [file pone.0287084.s001.pptx]

## Slide 1
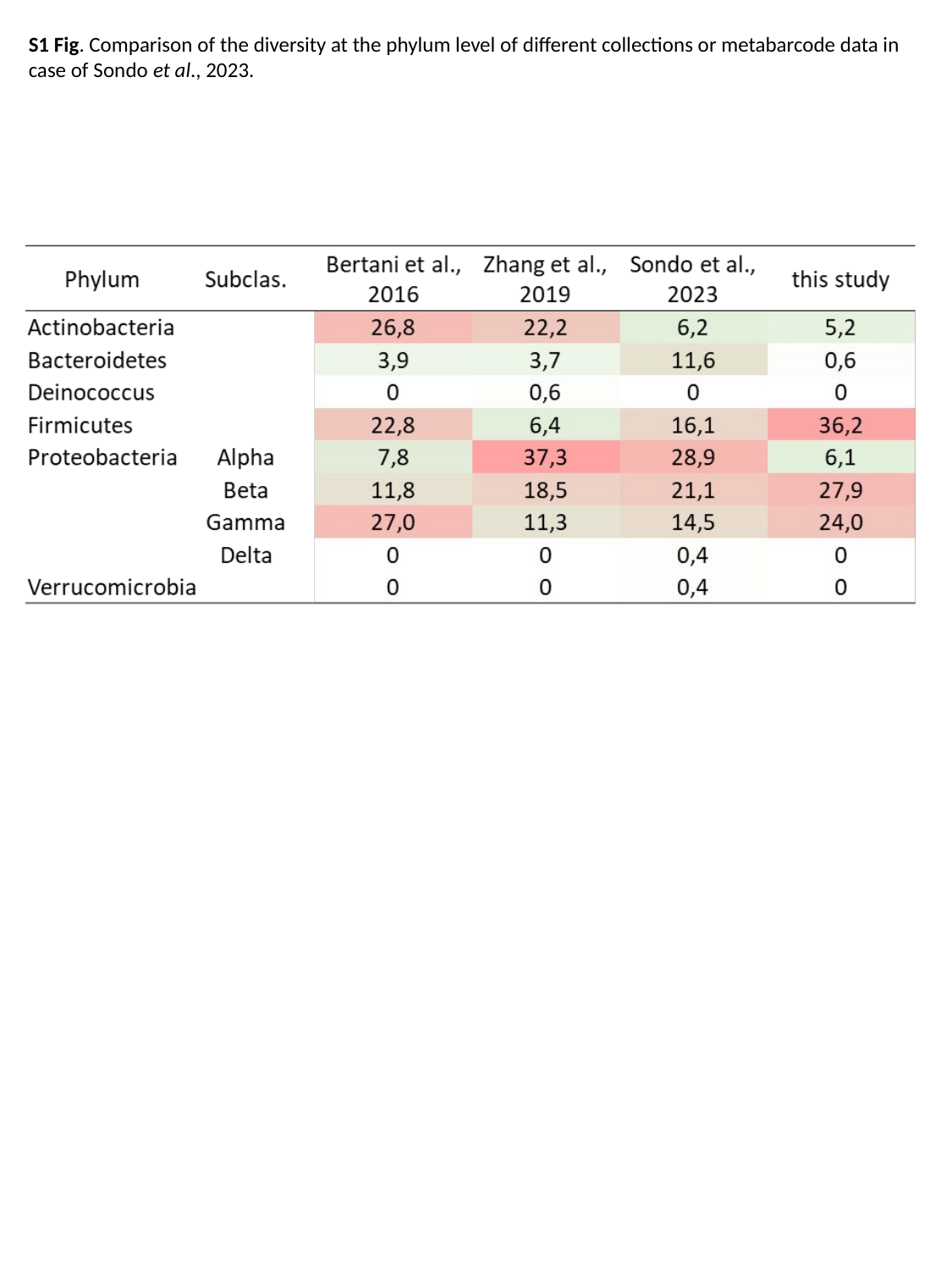

S1 Fig. Comparison of the diversity at the phylum level of different collections or metabarcode data in case of Sondo et al., 2023.
