# Supplementary material for: Diversity and plant growth promoting ability of rice root-associated bacteria in Burkina-Faso and cross-comparison with metabarcoding data
Source: PLoS One. 2023 Nov 30;18(11):e0287084. doi: 10.1371/journal.pone.0287084 (PMC10688718; doi:10.1371/journal.pone.0287084)
Supplement: S2 Fig — The principal component analysis of total root microflora (in bleu) versus endophytes (in red). (PPTX) [file pone.0287084.s002.pptx]

## Slide 1
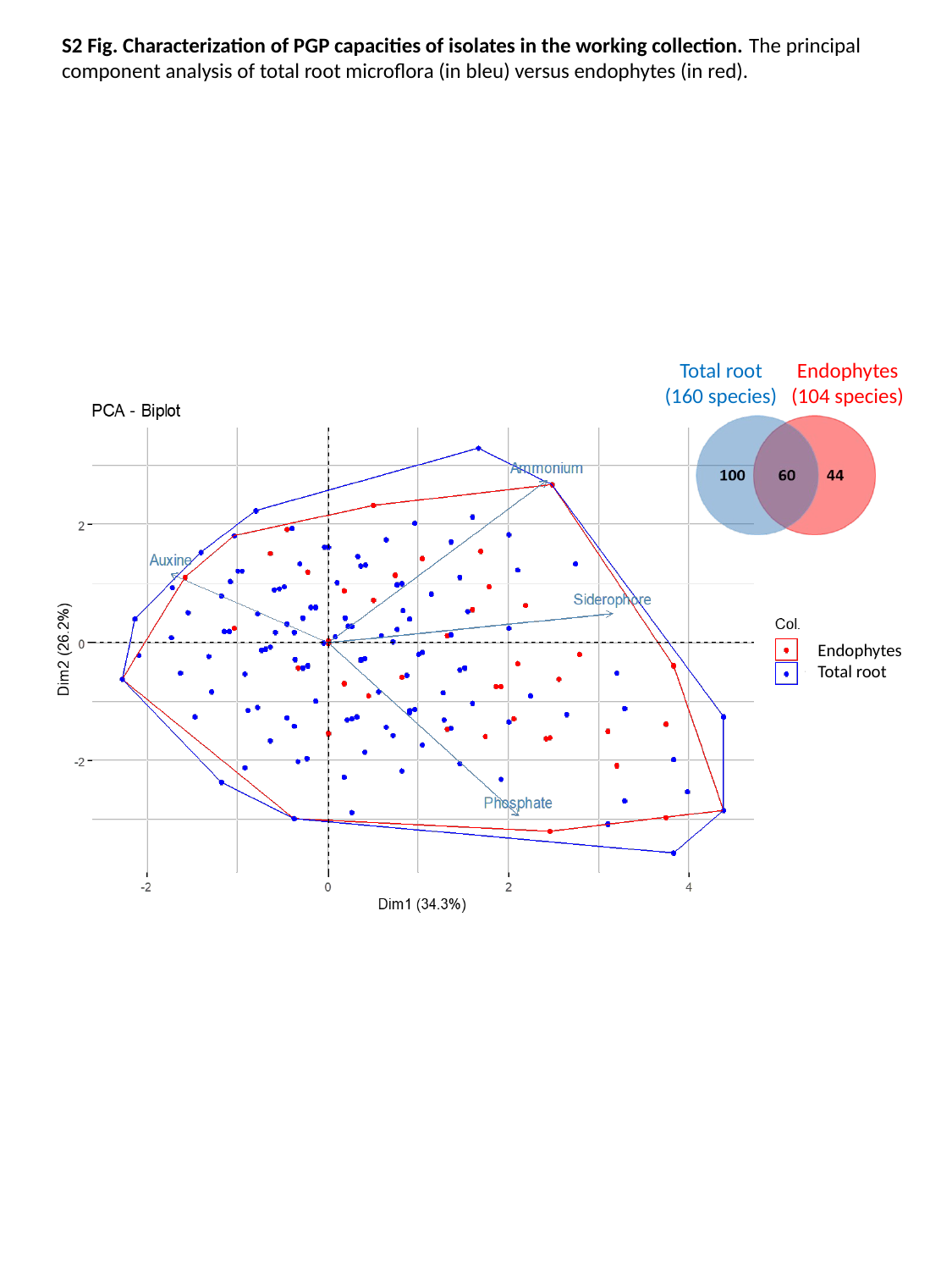

S2 Fig. Characterization of PGP capacities of isolates in the working collection. The principal component analysis of total root microflora (in bleu) versus endophytes (in red).
Total root
(160 species)
Endophytes (104 species)
Endophytes
Total root
