# Supplementary material for: Diversity and plant growth promoting ability of rice root-associated bacteria in Burkina-Faso and cross-comparison with metabarcoding data
Source: PLoS One. 2023 Nov 30;18(11):e0287084. doi: 10.1371/journal.pone.0287084 (PMC10688718; doi:10.1371/journal.pone.0287084)
Supplement: S3 Fig — The principal component analysis of root surface microflora (in bleu) versus endophytes (in red). (PPTX) [file pone.0287084.s003.pptx]

## Slide 1
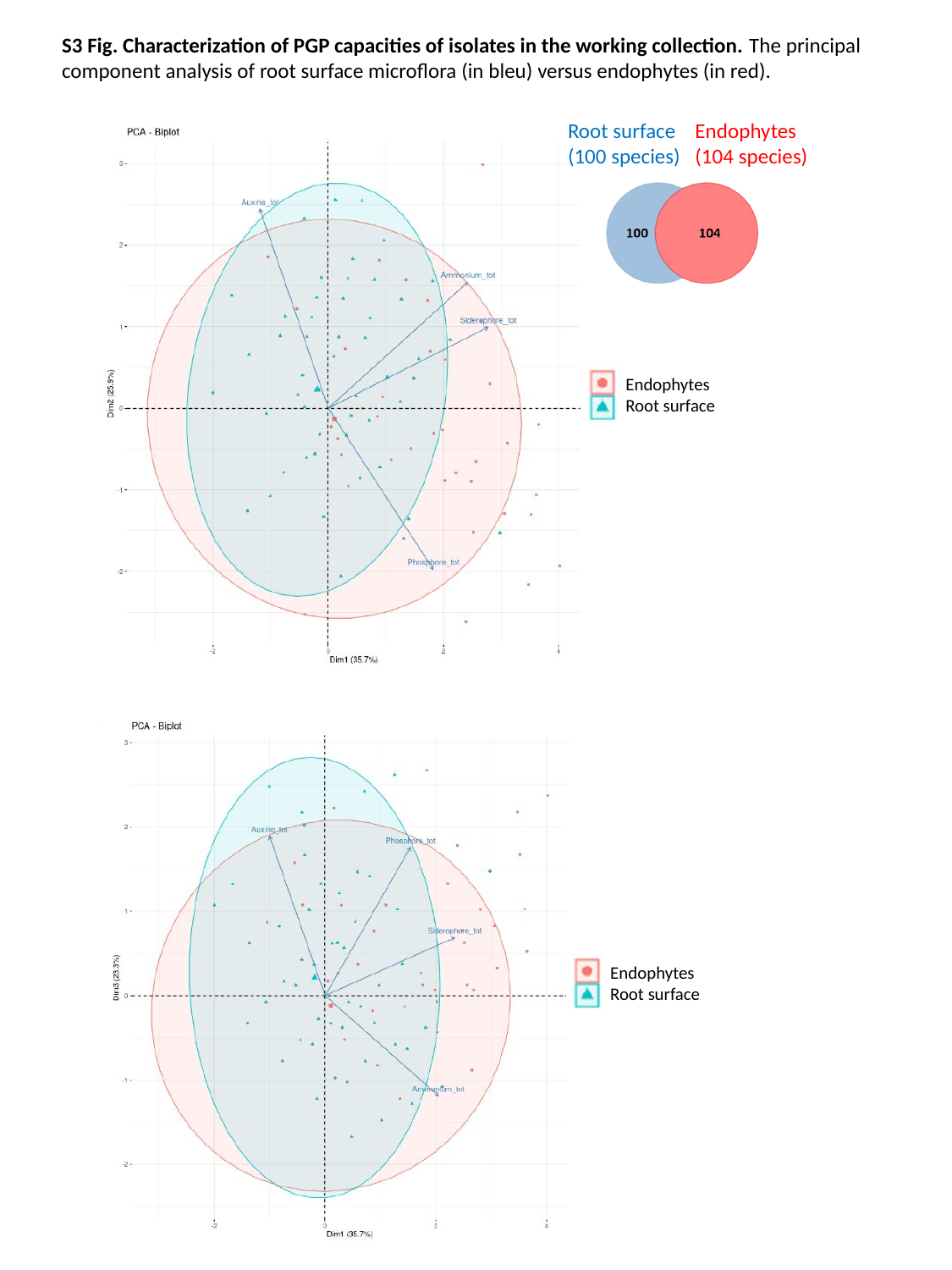

S3 Fig. Characterization of PGP capacities of isolates in the working collection. The principal component analysis of root surface microflora (in bleu) versus endophytes (in red).
Root surface
(100 species)
Endophytes
(104 species)
Endophytes
Root surface
Endophytes
Root surface
