# Supplementary material for: Diversity and plant growth promoting ability of rice root-associated bacteria in Burkina-Faso and cross-comparison with metabarcoding data
Source: PLoS One. 2023 Nov 30;18(11):e0287084. doi: 10.1371/journal.pone.0287084 (PMC10688718; doi:10.1371/journal.pone.0287084)
Supplement: S4 Fig — A, results of Wilcoxon test for comparison of the averages for each molecule independently according to the microflora groups; B, boxplot representations of PGP capacities for root surface and endophyte populations. (PPTX) [file pone.0287084.s004.pptx]

## Slide 1
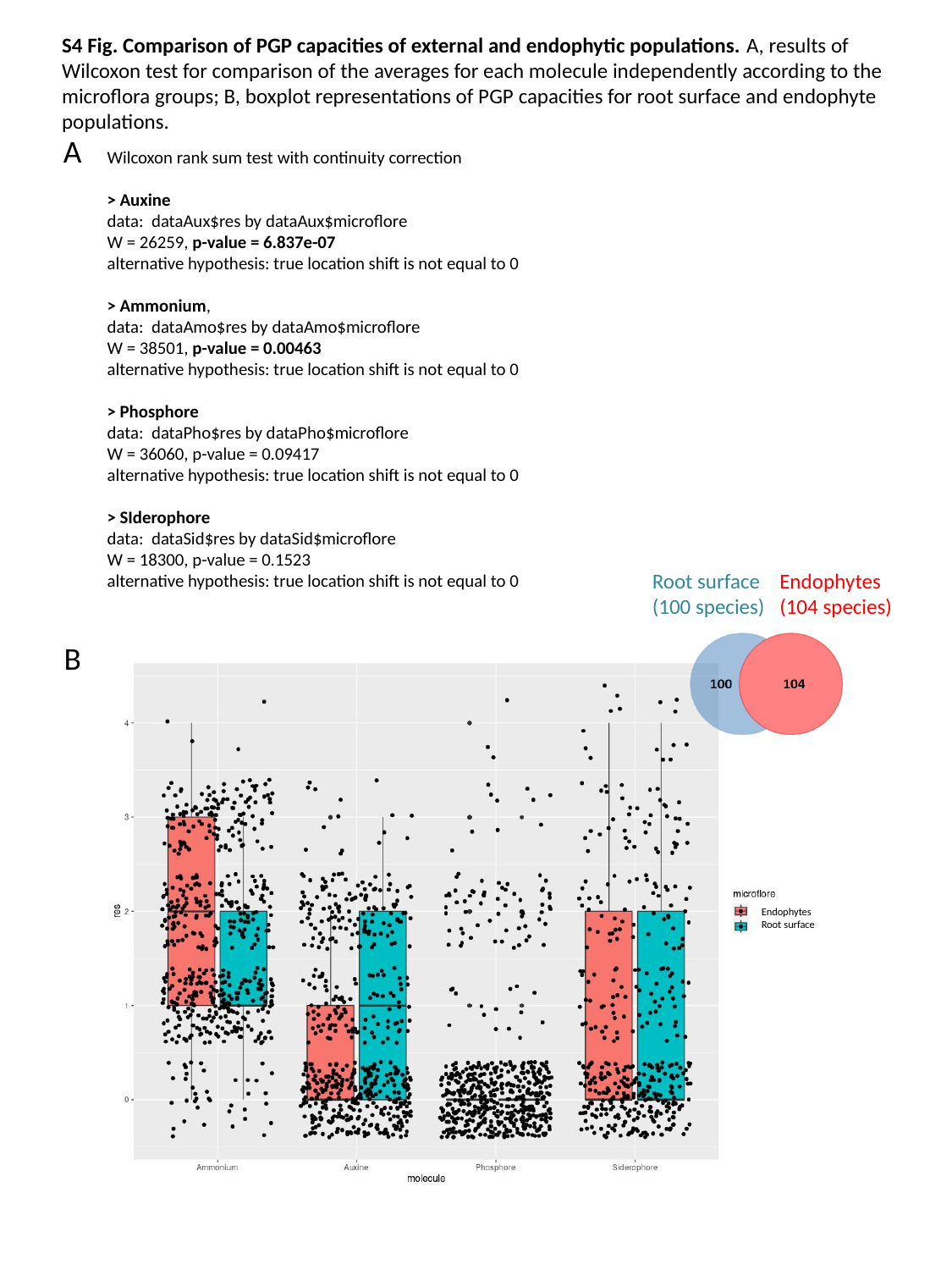

S4 Fig. Comparison of PGP capacities of external and endophytic populations. A, results of Wilcoxon test for comparison of the averages for each molecule independently according to the microflora groups; B, boxplot representations of PGP capacities for root surface and endophyte populations.
A
Wilcoxon rank sum test with continuity correction
> Auxine
data: dataAux$res by dataAux$microflore
W = 26259, p-value = 6.837e-07
alternative hypothesis: true location shift is not equal to 0
> Ammonium,
data: dataAmo$res by dataAmo$microflore
W = 38501, p-value = 0.00463
alternative hypothesis: true location shift is not equal to 0
> Phosphore
data: dataPho$res by dataPho$microflore
W = 36060, p-value = 0.09417
alternative hypothesis: true location shift is not equal to 0
> SIderophore
data: dataSid$res by dataSid$microflore
W = 18300, p-value = 0.1523
alternative hypothesis: true location shift is not equal to 0
Root surface
(100 species)
Endophytes
(104 species)
B
Endophytes
Root surface
