# Supplementary material for: Diversity and plant growth promoting ability of rice root-associated bacteria in Burkina-Faso and cross-comparison with metabarcoding data
Source: PLoS One. 2023 Nov 30;18(11):e0287084. doi: 10.1371/journal.pone.0287084 (PMC10688718; doi:10.1371/journal.pone.0287084)
Supplement: S5 Fig — Two rice varieties were tested, FKR64 (TS2) and Orylux 6. Size, plant length (cm), LW, leaves dry weight (g), RW, roots dry weight (g). (PPTX) [file pone.0287084.s005.pptx]

## Slide 1
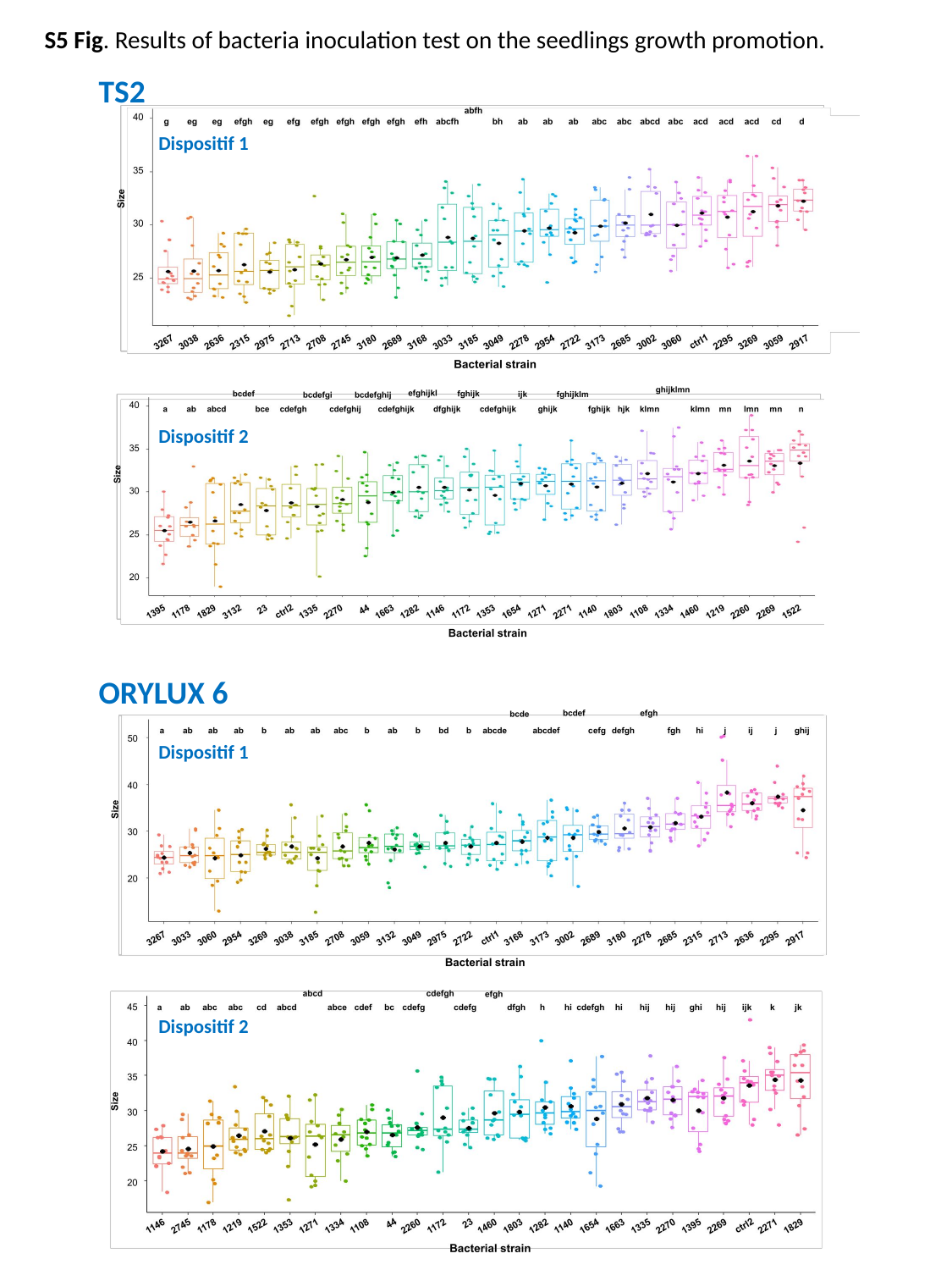

S5 Fig. Results of bacteria inoculation test on the seedlings growth promotion.
TS2
Dispositif 1
Dispositif 2
ORYLUX 6
Dispositif 1
Dispositif 2

## Slide 2
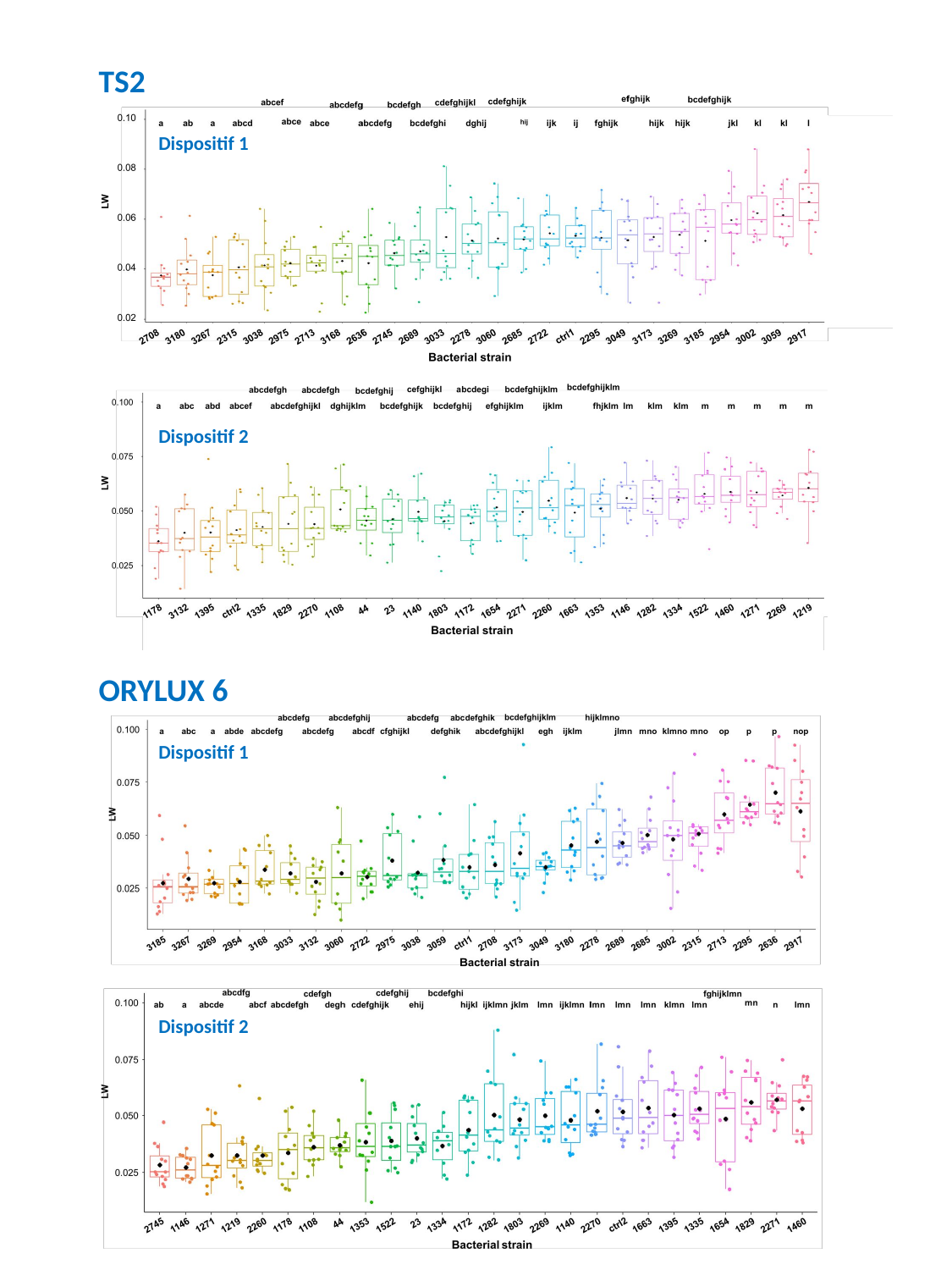

TS2
Dispositif 1
Dispositif 2
ORYLUX 6
Dispositif 1
Dispositif 2

## Slide 3
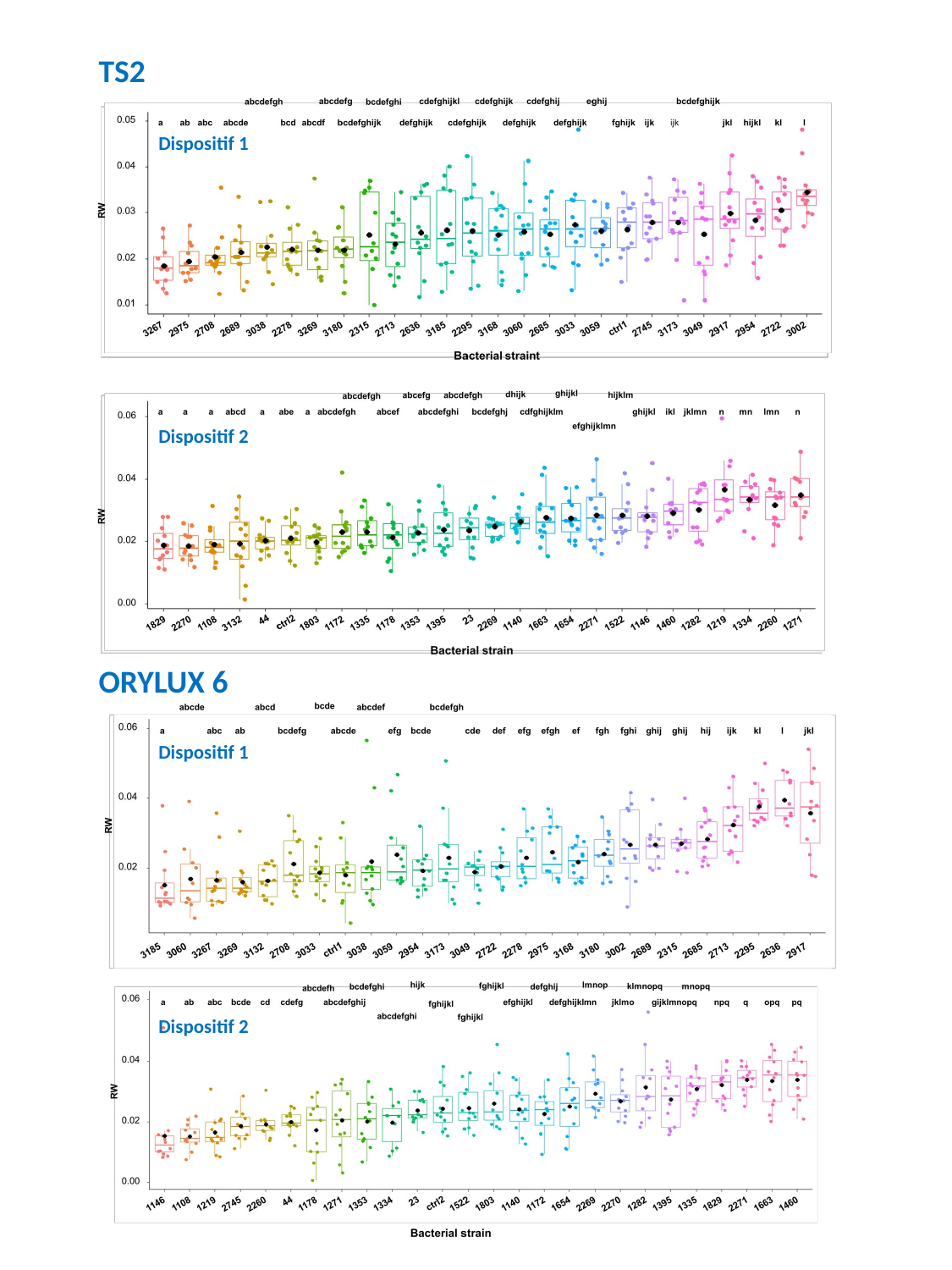

TS2
Dispositif 1
Dispositif 2
ORYLUX 6
Dispositif 1
Dispositif 2
