# Supplementary material for: Diversity and plant growth promoting ability of rice root-associated bacteria in Burkina-Faso and cross-comparison with metabarcoding data
Source: PLoS One. 2023 Nov 30;18(11):e0287084. doi: 10.1371/journal.pone.0287084 (PMC10688718; doi:10.1371/journal.pone.0287084)
Supplement: S6 Fig — CTRL1 and CTRL2, controls from dispositive one and two. (PPTX) [file pone.0287084.s006.pptx]

## Slide 1
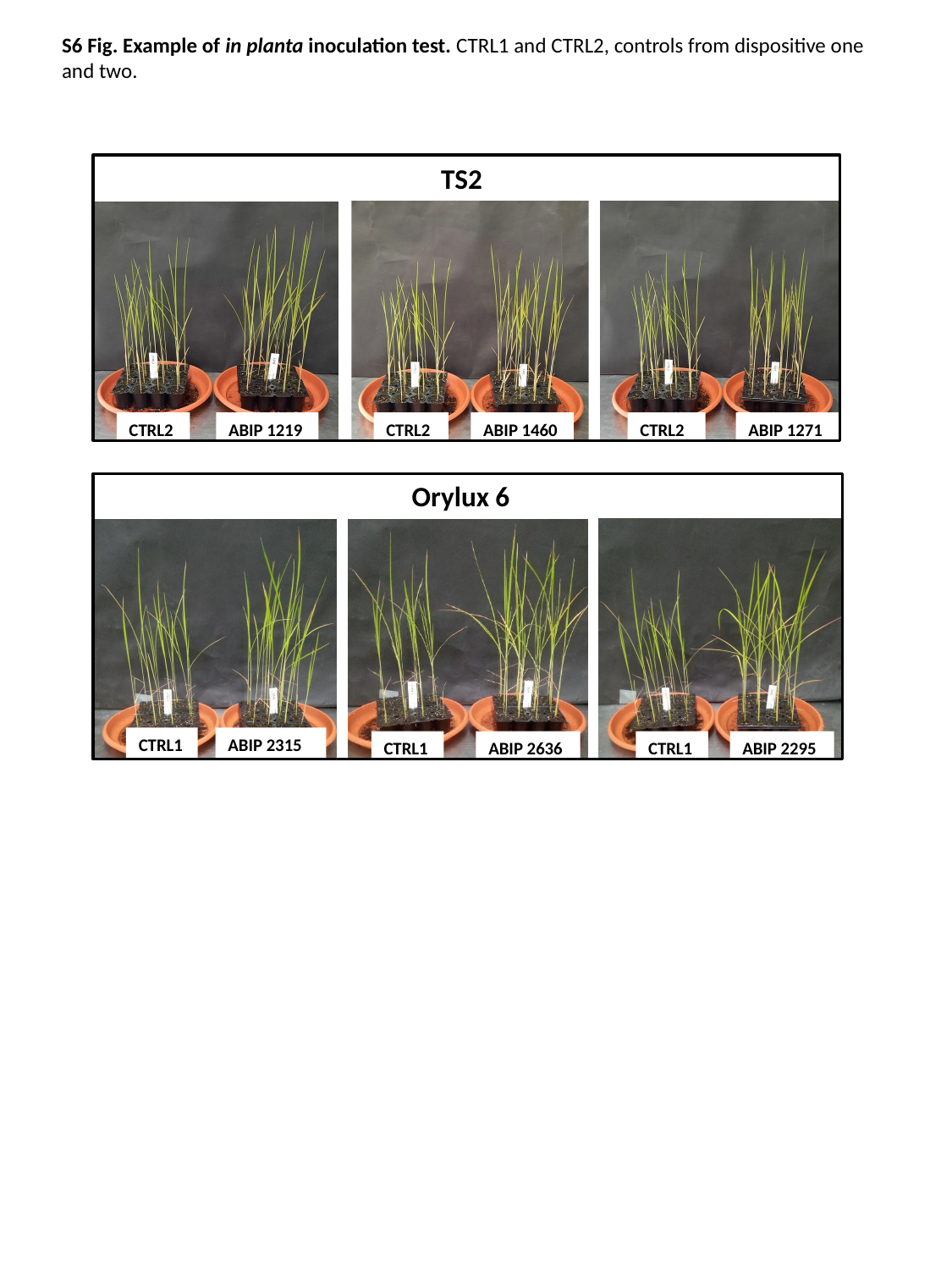

S6 Fig. Example of in planta inoculation test. CTRL1 and CTRL2, controls from dispositive one and two.
TS2
CTRL2
ABIP 1219
CTRL2
ABIP 1460
CTRL2
ABIP 1271
Orylux 6
CTRL1
ABIP 2315
CTRL1
ABIP 2636
CTRL1
ABIP 2295
